# A cross-sectional study on health behavior and quality of life among adults with non-communicable diseases in the urban field practice area of a teaching hospital, Kolkata

**DOI:** 10.51866/oa.562

**Published:** 2025-02-27

**Authors:** Shalini Pattanayak, Sinjita Dutta, Mausumi Basu, Subhra Samujjwal Basu, Sukanta Manna

**Affiliations:** 1 M.D. (Community Medicine), Department of Community Medicine, IPGME&R and SSKM Hospital, Kolkata, India. Email: s.subhra@gmail.com; 2 M.B.B.S, Department of Community Medicine, IPGME&R and SSKM Hospital, Kolkata, India.; 3 M.D. (Community Medicine), Department of Community Medicine, IPGME&R and SSKM Hospital, Kolkata, India.; 4 M.D. (Community Medicine), Department of Community Medicine, IPGME&R and SSKM Hospital, Kolkata, India.; 5 M.B.B.S, Department of Community Medicine, IPGME&R and SSKM Hospital, Kolkata, India.

**Keywords:** Diabetes, Health behaviour, Hypertension, Non-communicable diseases, Quality of life

## Abstract

**Introduction:**

Non-communicable diseases (NCDs) are considered the leading causes of death globally, accounting for 60% of all deaths. Measures such as stopping tobacco use, increasing physical activity, reducing alcohol consumption and improving diet can extend longevity and enhance the Quality of Life (QoL). This study aimed to assess the overall health behaviours and QoL of adults with NCDs residing in the urban field practice area of a teaching hospital in Kolkata and determine the association of their sociodemographic characteristics and health behaviours with their overall QoL.

**Methods:**

A cross-sectional study was conducted in the outpatient department of the Urban Primary Health Centre-81 among 106 adults recruited via consecutive sampling. Face-to-face interviews were conducted using a predesigned, pretested and structured schedule. Data were analysed using the Statistical Package for the Social Sciences version 25.0. Descriptive and inferential statistics were employed to interpret the data.

**Results:**

Among the participants, 45.3% were consuming tobacco, while only 6% were consuming alcohol following the diagnosis of NCDs. The majority (81%) undertook brisk walking, and 37% reported additional salt intake with meals. The overall perceived QoL was poor in 54.7% of the participants. The participants aged 41-60 years and the male participants had lower odds of having a poor QoL than their counterparts.

**Conclusion:**

More than half of adults with NCDs report a poor QoL. Extensive interventions are needed to raise awareness in the community regarding the potential benefits of lifestyle modifications following the diagnosis of any NCD and thereby improve the QoL of patients.

## Introduction

Heart disease, cancer, stroke, chronic respiratory diseases and diabetes are Non-Communicable Diseases (NCDs) considered the leading causes of death in India and other countries worldwide. Globally, these chronic conditions account for 60% of all deaths.^1^ In Southeast Asia, NCDs are responsible for 14.5 million deaths, and tobacco smoking directly contributes up to 14% of all NCD deaths among adults, which is expected to rise to 6.8 million in 2030 in low- and middle-income countries in the absence of robust action.^1,2^ According to the World Health Organization (WHO), NCDs are the leading causes of death worldwide, responsible for 71% of the total number of deaths each year. The top four NCDs with the highest number of deaths are cardiovascular diseases (17.9 million deaths annually), cancers (9.0 million deaths), respiratory diseases (3.9 million deaths) and diabetes (1.6 million deaths).^3,4^ NCDs are of increasing concern for society and national governments as well as globally due to their high mortality rate.^5^ The main risk factors contributing to NCDs include unhealthy diet, physical inactivity, tobacco use and alcohol misuse. The National Programme for Control of Non-Communicable Diseases (NP-NCD) was launched by the government of India to strengthen health systems.^6,7^ Since NCDs are influenced by modifiable behaviours, measures such as adopting a healthier lifestyle, including stopping smoking, increasing physical activity, eliminating tobacco use and heavy alcohol consumption and improving diet, can extend longevity, reduce the recurrence of an adverse event and enhance the quality of life (QoL). Various studies on the QoL of patients with NCDs such as diabetes have reported that diabetes has a negative impact on the QoL.

In Ethiopia, Reba et al. reported a low mean score of the overall health-related quality of life (HRQoL) and its domain among patients diagnosed with type 2 diabetes mellitus, which was affected by their educational level, marital status, occupation, duration of diabetes mellitus and presence of diabetes mellitus complications.^8^ Zheng et al. found that older adult patients with hypertension in China had a lower HRQoL than the general population.^9^ Similarly, Liu et al. showed a positive relationship between the number of NCDs and the HRQoL of older people.^10^

Population-level information about lifestyle changes among people diagnosed with NCDs is lacking. Therefore, the current study aimed to assess the health behaviours and QoL of adults with NCDs in the urban field practice area of a teaching hospital in Kolkata.

## Methods

### Study type, design and setting

The study adopted a descriptive, observational, cross-sectional design and was conducted in the outpatient department (OPD) of the Urban Primary Health Centre (UPHC) under KMC Ward-81, Chetla, Kolkata, located in the urban field practice area of the Institute of Post Graduate Medical Education and Research (IPGME&R) and Seth Sukhlal Karnani Memorial (SSKM) Hospital, Kolkata.

### Study duration

The study was performed for approximately 2 months, from April to May 2023.

### Study participants

**Inclusion criteria**

All patients more than 18 years of age, belonging to any sex, diagnosed with any NCD since >1 year ago and attending the OPD of the UPHC-81 were included in the study. In cases of NCDs, the QoL may not be affected immediately after the diagnosis especially if diagnosed at an earlier stage. Similarly, changes in health behaviours after the diagnosis of NCDs do not take place instantly. Thus, we included patients with NCDs with a duration of at least 1 year in the current study.

**Exclusion criteria**

Patients who were mentally incapable of providing the information required for the study, pregnant and lactating women and patients who did not provide informed written consent for participation were excluded from the study.

### Sample size estimation and sampling technique

The sample size was calculated using Cochran’s formula: Z^2^xpq/l^2^, where p is the proportion of study participants with both diabetes and hypertension (50%); q is calculated as 1-p; Z is the standard normal deviation (1.96); and d is the absolute error rate (10%). The sample size was calculated as approximately 96, with a 95% Confidence Interval (CI). Given a 10% nonresponse rate, the final sample size estimated was approximately 106. The consecutive sampling technique was employed to select participants.

### Study tools and technique

A predesigned, pretested and structured schedule, comprising the following sections, was used:

i) sociodemographic characteristics, ii) health behaviours iii) Quality of Life (QoL) evaluated using the WHOQOL-BREF.^11^

The Out Patient Department (OPD) of the UPHC-81 operates from Monday to Saturday every week, from 9 am to 2 pm. All patients aged ≥18 years, belonging to any sex, attending the OPD of the target centre during 1 month of the data collection period and fulfilling the eligibility criteria were included in the study. Informed written consent was obtained from participants prior to conducting the study. Face-to-face interviews were conducted after the OPD consultation using the predesigned, pretested and structured schedule, which was initially constructed in English, later translated into Bengali by a Bengali language expert and Hindi by a Hindi language expert and later retranslated into English by respective language experts to ensure content and construct validity. The face-to-face interviews were conducted at the NCD OPD for better understanding and establishing rapport with participants. Nine eligible study participants were interviewed consecutively for 3 days a week for 1 month to achieve the desired sample size of 106.

### Study variables

The dependent variable was the QoL, evaluated using the WHOQOL-BREF This pre-validated scale was developed by the WHO for assessing the QoL. It evaluates individuals’ perception of their position in life in the context of the culture and value systems in which they live and in relation to their goals, expectations, standards and concerns. The scale produces a multidimensional profile of scores across six domains and 24 sub-domains of the QoL. Responses are assigned according to their corresponding predefined scores ranging from 1 to 5, signifying the worst to the best possible health status for all questions, except for questions 3, 4 and 26, where the point values are reversed. The score is calculated for each of the four health domains by taking the average for each domain and multiplying it by 4. The domain scores are transformed into a scale ranging from 0 to 100, where 100 indicates the highest QoL and 0 indicates the lowest QoL, using the following formula: transformed score= (domain score-4) × (100/16). There is no defined cut-off point for the QoL in this scale. In this study, the first two questions in the WHOQOL-BREF were taken together for the analysis of the perceived QoL.

The independent variables were the sociodemographic characteristics and health behaviours of participants. The sociodemographic characteristics included age (in completed years), sex, religion, caste, socioeconomic status, highest level of education attained, occupation, marital status and any family history of NCDs. Health behaviours were assessed based on current tobacco and alcohol consumption, brisk walking and salt intake. Each domain was categorised into two divisions. For example, current tobacco intake and current alcohol intake were categorised as ‘yes’ or ‘no’ for each item; participants responding ‘yes’ were scored as ‘1’ and ‘no’ as ‘0’. Similarly, those who undertook brisk walking were scored as ‘1’ and those who did not as ‘0’. The scores obtained for each domain were added, and an overall median score was calculated to assess the overall health behaviours of participants.

### Statistical analysis

Data were tabulated using Microsoft Office Excel 2021 and analysed utilising the Statistical Package for the Social Sciences version 25.0 (Armonk, NY: IBM Corp., 2017). Descriptive and inferential statistics were used to interpret the data as applicable. Descriptive statistics were represented by means with standard deviations, frequencies and percentages. Individuals with a total mean score of 50% and above were classified as having a good QoL and those with a total mean score of less than 50% as having a poor QoL. Univariate regression analysis was performed to identify the association of the sociodemographic characteristics and health behaviours of participants with their QoL. All variables with a P-value of <0.2 in the univariate logistic regression analysis were considered biologically plausible and included in the multivariable model to check for model fitness after checking for multi-collinearity (variance inflation factor of >10 and tolerance value of <0.1). A P-value of <0.05 was considered statistically significant.

### Ethical considerations

Anonymity and confidentiality of the data were maintained throughout the study. Informed written consent was obtained from eligible study participants. A research proposal was submitted, and clearance was obtained from the Institutional Ethics Committee (IEC) of the IPGME&R and SSKM Hospital, Kolkata (**IPGME&R/IEC/2023/440**).

### Operational definitions

NCDs were defined as diseases that tend to have a long duration and arise from a combination of genetic, physiological, environmental and behavioural factors. The NCDs considered under the NPNCD (National Programme for Non-Communicable Diseases) are hypertension, diabetes, cardiovascular diseases (e.g., heart attack and stroke), cancers, chronic respiratory diseases (e.g., chronic obstructive pulmonary disease and asthma), non-alcoholic fatty liver disease and chronic kidney disease.^12^ In the current study, individuals more than 18 years of age diagnosed with one of the above-mentioned NCDs were included.

## Results

Among the study participants, 53.5% were women, and 38% were aged 41-60 years. Most of the participants followed Hinduism (86.8%). Approximately 48% belonged to the general caste, while 71.7% had a lower- to-middle (class IV) socioeconomic status (as per the Modified B.G. Prasad Scale, updated in October 2022). Almost 93% of the participants were married; only 12.1% completed secondary education and above; and 40.2% were employed (Table 1). Around 81% had a family history of any NCD; among them, the most common NCD was hypertension (54.7%), followed by diabetes mellitus (16.7%) and both diabetes mellitus and hypertension (12.7%).

**Table 1:** Sociodemographic characteristics of the participants (N=106).

No.	Sociodemographic characteristic	n (%)
1.	**Age (in completed years)**	
	18-40	35 (32.7)
	41-60	41 (38.3)
	≥61	30 (28.0)
2.	**Sex**	
	Male	49 (45.8)
	Female	57 (53.3)
3.	**Religion**	
	Hindu	92 (86.0)
	Others	14(14.0)
4.	**Caste**	
	General	51 (47.7
	Others	55 (52.3)
5.	**Educational level**	
	Illiterate	34 (31.8)
	Primary and middle school	59 (55.1)
	Secondary school and above	13 (12.1)
6.	**Occupation**	
	Unemployed	39 (36.4)
	Employed	43 (40.2)
	Housewife	24 (22.4)
7.	**Family history of any non-communicable disease**	
	Yes	88 (83.0)
	No	18 (17.0)
8.	**Marital status**	
	Married	99 (93.4)
	Widowed	7 (6.6)
10.	**Socioeconomic status (as per the Modified B.G. Prasad Scale, October 2022)**	
	Class II (Upper-to-middle)	2 (1.9)
	Class III (Middle)	11 (10.4)
	Class IV (Lower-to-middle)	76 (71.7)
	Class V (Lower)	17 (16.0)

All family members (100%) of the participants were receiving treatment for their respective NCDs following the diagnosis. The majority (55%) had hypertension, followed by diabetes mellitus (38%) and both diabetes mellitus and hypertension (7%). All participants (100%) diagnosed with NCDs were receiving treatment and were registered under the NPNCD, India. Only one (0.7%) male patient had a history of a cardiac event 6 months prior to the study, following which he was under treatment and regular follow-ups at the time of the study. Among the participants, 45.3% were still consuming any form of tobacco (smoking/ smokeless) following the diagnosis of any NCD, while only nine (6%) were still consuming alcohol. Of the participants who were still consuming any form of tobacco after the diagnosis of NCDs, the most common form was *bidi* (n=14), followed by *gurakhu* (n = 8). Nearly 81% were undertaking brisk walking. Almost 37% still consumed additional salt with their meals. All participants consumed fruits, among whom only two (1.3%) consumed such regularly (7 days a week). All participants consumed cooked vegetables 7 days a week.

The overall perceived QoL was ‘good’ and ‘poor’ in 45.3% and 54.7% of the participants, respectively.

The social relations domain (domain 3) had the highest mean score (82.8 ± 18.4), while the physical health domain (domain 1) had the lowest mean score (46.6 ± 9.4). Hence, the physical health domain of the QoL was the most negatively affected domain after the diagnosis of NCDs among the participants (Table 2).

**Table 2:** Distribution of the mean (± SD) scores for each of the four domains of the QoL among the participants (N=106)

Domain	Domain name	Mean	SD
Domain 1	Physical health	48.4	8.2
Domain 2	Psychological health	48.5	4.7
Domain 3	Social relations	84.5	16.1
Domain 4	Environment	50.8	5.8

The participants aged 41-60 years (aOR=0.290, 95% CI=0.09-0.85; P=0.025), male participants (aOR=0.295, 95% CI=0.12-0.72; P=0.007) and illiterate participants (aOR=0.154, 95% CI=0.03- 0.73; P=0.019) had lower odds of having a poor QoL than their counterparts (Table 3). The omnibus test for model coefficients yielded a significant value (P=0.003), while the Hosmer-Lemeshow goodness-of-fit test revealed a non-significant value (P=0.279), suggesting a good fit for the model.

**Table 3:** Multivariable binary logistic regression analysis of the association between the sociodemographic characteristics and overall perceived QoL among the participants (N=106).

No.	Sociodemographic characteristic	Poor QoL (n_1_)		
		n (%)	aOR (95% CI)	P-value
1.	**Age (in completed years)**			
	18-40	35 (33.0)	0.53 (0.17-1.58)	0.25
	41-60	41 (38.7)	0.29 (0.10-0.85)	0.02
	≥61	30 (28.3)	Ref.	-
2.	**Sex**			
	Male	49 (45.8)	0.30 (0.12-0.72)	0.007
	Female	57 (53.3)	Ref.	
3.	**Highest level of education attained**			
	Illiterate	34 (31.8)	0.15 (0.03-0.73)	0.01
	Primary and middle school	59 (55.1)	0.30 (0.07-1.29)	0.10
	Secondary school and above	13 (12.1)	Ref.	-
4.	**Occupation**			
	Unemployed	39 (36.4)	2.95 (0.89-9.75)	0.07
	Employed	43 (40.2)	2.49 (0.76-8.10)	0.13
	Homemaker and others	24 (22.4)	Ref.	-

*Reference category=last (good QoL),* Model fitness information: Cox and Snell R-square=0.183, Nagelkerke R-square=0.245, Omnibus test for model coefficients: P=0.003, which was statistically significant, Hosmer-Lemeshow goodness-of-fit test: P=0.279, which was not statistically significant n1=58, indicating the number of participants with a poor overall perceived QoL

The health behaviours following the diagnosis of NCDs, which included the consumption of additional salt with meals and tobacco, were biologically plausible in the univariate model, but in the multivariate model, no significant association was found between the perceived QoL and consumption of additional salt and tobacco (Table 4). In this case, the omnibus test for model coefficients and Hosmer-Lemeshow goodness-of-fit test did not yield significant values (P=0.153 and 0.983, respectively).

**Table 4:** Multivariable binary logistic regression analysis of the association between the health behaviours after the diagnosis of NCDs and overall perceived QoL among the participants (N=106).

No.	Health behaviour following the diagnosis of NCDs	Poor QoL (n_1_)		
		n (%)	aOR (95% CI)	P-value
1.	**Current consumption of any form of tobacco**			
	Yes	48 (45.3)	1.76 (0.79-3.92)	0.16
	No	58 (54.7)	Ref.	
2.	**Current consumption of additional salt with meals**			
	Yes	39 (36.8)	1.50 (0.66-3.43)	0.33
	No	67 (63.2)	Ref.	

Reference category=last (good QoL), Model fitness information: Cox and Snell R-square=0.35, Nagelkerke R-square=0.46, Omnibus test for model coefficients and Hosmer—Lemeshow goodness-of-fit test: P=0.153 and 0.983, respectively, which were not statistically significant n1=58, indicating the number of participants with a poor overall perceived QoL

## Discussion

### Sociodemographic characteristics

In the study conducted by Oliveira-Campos et al. among adults more than 18 years of age with NCDs, who were mostly women (73.8%), the most commonly affected domain in the SF-36 questionnaire was vitality (energy and fatigue).^13^ Similarly, our study recruited adults more than 18 years of age, who were mostly women (53.5%), and used the WHOQOL- BREF to assess the QoL. The findings showed that the physical health domain (domain 1) was the most frequently affected out of the four domains in the scale. The larger proportion of women in both studies reflects the fact that women were predominantly present in their homes during the data collection period, and their involvement in regular household chores may have contributed to the findings observed in both studies.

### Health behaviours

In the survey of the risk factors of NCDs in urban slums conducted by Anand et al. in Faridabad (Haryana), *bidi* was found to be the predominant form of smoked tobacco.^14^ While 26% of men reported consuming alcohol in the past 1 year, no women did, and only 7.9% and 5.4% of men and women, respectively, consumed five or more servings of fruits and vegetables per day. This study used the STEPS manual as a study tool. In the present study, the WHOQOL-BREF was used, along with a structured interview schedule to assess the QoL and health behaviours of the participants after the diagnosis of NCDs. The most common form of tobacco consumed was *bidi*. This may be because the procurement of *bidis* was easier and cheaper, thereby making it convenient for consumption, with a minimal effect on the overall household expenses. All participants consumed fruits, among whom only two (1.3%) consumed such regularly (7 days a week). All participants consumed cooked vegetables 7 days a week. Additionally, around 81% had a family history of any NCD. Approximately 55% had hypertension; 38%, diabetes mellitus; and 7%, both diabetes mellitus and hypertension. In contrast, Gong et al. reported that the prevalence of NCDs among older adults was 75.1%, with three or more NCDs accounting for 38.6%.^15^

### QoL

Al-Noumani et al. examined the predictors of the HRQoL, including health literacy, social support, patient-physician relationship and medication adherence, among patients with NCDs.^16^ Their findings revealed that participants’ mean age was 56 years, and most were women (52%), were unemployed (58%) and either received no education or had a low educational level (65%). Medication adherence and social support were consistently found as significant predictors for most dimensions of the HRQoL. In our study, most participants were women, were housewives and received middle-school education. The WHOQOL- BREF was used to assess the QoL of the participants. The participants aged 41-60 years (aOR=0.290, 95% CI=0.09-0.85; P=0.025), male participants (aOR=0.295, 95% CI=0.12- 0.72; P=0.007) and illiterate participants (aOR=0.154, 95% CI=0.03-0.73; P=0.019) had lower odds of having a poor QoL than their counterparts. According to Wang et al., a low socioeconomic status was associated with continuing or initiating physical inactivity and continuing smoking after NCD diagnosis compared to a high socioeconomic status.^17^ In our study, 45.3% of the participants were still consuming any form of tobacco (smoking/ smokeless) following the diagnosis of NCDs. This significant association may be due to a lack of awareness or motivation and the low socioeconomic status of the participants.

### Limitations

The current study was conducted in the OPD of a selected slum area located in the urban field practice area of one tertiary care institute. Hence, the findings may not represent the overall health behaviours and perceived QoL following the diagnosis of any NCD among the adult population. Some health behaviours such as the regularity of physical activity, sleep patterns, social life and adherence to medication could not be conveniently assessed in the OPD, as the study relied on self-reported data, which may also be subjected to social desirability bias.

## Conclusion

More than half of adults have hypertension, followed by diabetes mellitus, while few have both diabetes mellitus and hypertension. Almost half have a poor QoL following the diagnosis of any NCD. The physical health domain of the QoL is most negatively affected after the diagnosis of any NCD. Individuals aged 41-60 years, men and illiterate patients have lower odds of having a poor QoL than their counterparts. However, the health behaviours of adults following the diagnosis of any NCD, including the consumption of additional salt with meals and tobacco, are not significantly associated with their perceived QoL. These findings highlight the need for interventions aiming to increase awareness in the community regarding the potential benefits of lifestyle and behavioural modifications following the diagnosis of any NCD. The role of health workers such as accredited social health activists (ASHAs) becomes imperative in this regard. ASHAs should be mobilised to follow up on the dietary adherence and addiction pattern of patients visiting OPDs. Moreover, nutritional interventions, physical training and meditation camps may be arranged in the locality with the help of various NGOs and peer groups to maintain constant motivation and support for lifestyle changes. Strengthening healthcare systems, focusing on the early detection and treatment of NCDs and implementing robust surveillance measures are required, along with bolstering screening, diagnostic and treatment services in both urban and rural areas. Collaborations with different ministries, multisectoral approaches and strengthening of referral systems with involvement/training of grassroot-level workers should be considered to enhance population-based screening at frequent intervals. Screening programmes at the population level can substantially contribute to reducing the prevalence and impact of NCDs, leading to healthier communities and thus paving the way for a significant reduction in the global burden of these diseases. Effective treatment can help control symptoms, prevent complications, reduce costly interventions and hospitalisations and ultimately enhance overall patient well-being.

## Data sharing statement

Data are available upon request.

**How does this paper make a difference in general practice?**
This paper is expected to make a difference in general practice by:

- Incorporating lifestyle and behavioral modifications is needed right from the diagnosis of NCDs.
- The QoL was mostly affected in the age group of 41-60 years; thus, lifestyle and behavioral change programs should be tailored keeping in mind the job patterns, available time and workplace environment.
- Illiteracy can be detrimental to the adaptation of healthy lifestyle, so culture specific messages for dissemination of proper information is needed.
- The assumption that QoL will make people with NCDs change health behavior may not always work, hence specific interventions are needed for bringing the desired lifestyle changes in people with NCDs.
